# Supplementary material for: Arsenic Trioxide and Venetoclax Synergize against AML Progenitors by ROS Induction and Inhibition of Nrf2 Activation
Source: Int J Mol Sci. 2022 Jun 12;23(12):6568. doi: 10.3390/ijms23126568 (PMC9223383; doi:10.3390/ijms23126568)
Supplement: Supplementary file 1 [file ijms-23-06568-s001.zip › ijms-1739056-supplementary.pdf]

SUPPLEMENTAL INFORMATION

1> **Supplemental Figure S1 & Figure Legend for Supplemental Figure S1** (Page 2) Synergistic effects of ATO and VEN on cell viability in AML blasts.

2> **Supplemental Tables** (Page 3 to Page 7)

+ **Supplemental Table S1.** Patient characteristics of primary AML samples

+ **Supplemental Table S2.** Primer sequences used for qPCR analysis

+ **Supplemental Table S3.** Target sequences of siRNAs

+ **Supplemental Table S4.** List of antibodies used for IF, IP and IB analysis

SUPPLEMENTAL FIGURE S1 & FIGURE LEGEND FOR SUPPLEMENTAL FIGURE S1

Figure S1. Effect of VEN plus ATO combinatorial treatment on growth of primary CD34+CD38− AML progenitor cells in vitro. Cells were treated for 48 h with control vehicle, individual drugs or with the 2-drug combination at constant drug ratios, on the basis of previously established IC_{50} values, followed by measurement of cell proliferation. Drug synergy was analyzed using the CalcuSyn program and is shown in Figure 3F (isobologram).

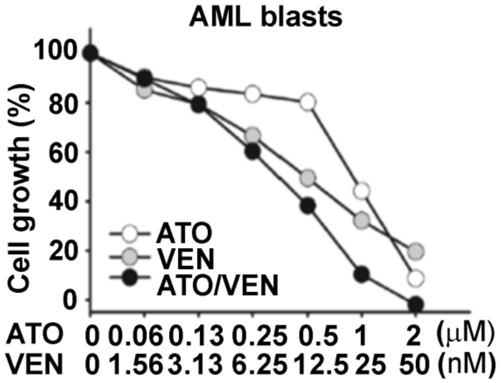

SUPPLEMENTAL TABLES

Table S1. Patient characteristics of primary AML samples

Sample ID	Sample Type	Disease Status	Response	Cytogenetic	Gene Mutation	WBC	Blasts in PB	Blasts in BM
AML pool samples								
AML001	PB	Untreated	N/A	Normal	FLT-3 ITD Pos., NPM1 Neg., CEBPA Pos.	76.8	84	75
AML002	BM	Induction Failure	N/A	Normal	FLT-3 ITD Pos., NPM1 Pos.	4.2	91	90
AML003	PB	Untreated	N/A	Normal	FLT-3 Neg., NPM1 Pos.	5.7	72	
AML004	PB	Untreated	N/A	Normal	FLT-3 Neg., NPM1 Neg.	29.0	91	
AML005	PB	Untreated	N/A	Normal	FLT-3 Neg., NPM1 Neg.	12.6	69	
VEN-sensitive and VEN-resistant samples								
AMLS01	BM	New	CR	Stemline:	TP53;U2AF1;ASXL	89.4	6	73

		diagnosis		46,XY,del(17)(p11.2)[16] Constitutional Cell Line: 46,XY[4] FISH: Positive for loss of TP53	1;RUNX1;FLT3 (ITD)			
AMLS02	BM	New diagnosis	CR	46,XX[20] FISH: No assay-specific abnormalities detected	DNMT3A;NPM1;TET2	22.1		2
AMLS03	BM	New diagnosis	CR	Normal	SF3B1, NPM1, NRAS	12.6		20
AMLR01	PB	Progressing/ high	Refractory	FISH: No assay-specific	Inv 3, FLT3 TKD, PTPN11	98.6	89	79.6

		grade		abnormalities detected				
AMLR02	PB	Progressing/ high grade	Refractory		FLT3-ITD	58.9	69	
AMLR03	BM	Progressing/ high grade	Refractory	Stemline: 46,XY,t(9;11)(p21.3;q23.3)[3]<br>Sideline 1: 49,sl,+der(9)t(9;11),+20,+21[12]<br>Sideline 2: 51,sdl1,+3,+21[5]<br>FISH:<br>Positive for KMT2A rearrangement with or without		0.8	75	72

				concurrent gain of 3'KMT2A				

Table S2. Primer sequences used for qPCR analysis

No	Primer name	Sequence
1	NQO-1-F	**5’ GCCGCAGACCTTGTGATATT 3’**
2	NQO-1-R	**5’ TTTCAGAATGGCAGGGACTC 3’**
3	HO-1-F	**5’ ATGGCCTCCCTGTACCACATC 3’**
4	HO-1-R	**5’ TGTTGCGCTCAATCTCCTCCT 3’**
5	GAPDH-F	**5’ CCCCTTCATTGACCTCAACTACAT 3’**
6	GAPDH-R	**5’ CGCTCCTGGAAGATGGTGA 3’**

Table S3. Target sequences of siRNAs

No	Name	Target Sequence
1	siAKT	**CAUCACACCACCUGACCAA**<br>**ACAAGGACGGGCACAUUAA**<br>**CAAGGGCACUUUCGGCAAG**<br>**UCACAGCCCUGAAGUACUC**
2	siNrf2	**GCAUGCUACGUGAUGAAGA**<br>**CUCCUACUGUGAUGUGAAA**<br>**GUGUCAGUAUGUUGAAUCA**

Table S4. List of antibodies used for IF, IP and IB analysis

No	Antibody name	Information
1	Anti-Tubulin antibody	Clone# B-7, Cat# sc-5286, Santa Cruz
2	Anti-Actin antibody	Clone# C4, Cat# sc-47778, Santa Cruz
3	Anti-p-AKT(Ser473) antibody	Cat# 9271, Cell Signaling
4	Anti-PARP antibody	Cat# 9542, Cell Signaling
5	Anti-AKT antibody	Cat# 9272, Cell Signaling
6	Anti-Nrf2 antibody	Cat# 137550, Abcam
7	Anti-Keap1 antibody	Cat# SAB2501696, Sigma
8	Anti-Ubiquitin antibody	Cat# 07-375, Millipore
